# Sublethal Clothianidin Exposure Impairs Development, Thyroid Hormones, Locomotion and Predation in *Fejervarya cancrivora* from Rice Paddy Ecosystems

**DOI:** 10.3390/toxics14030243

**Published:** 2026-03-11

**Authors:** Joko Pilianto, Amr Abou El-Ela, Asim Munawar, Xiangfen Zhang, Dun Wang, Abid Ali Soomro, Naved A. Ansari, Wenwu Zhou, Zengrong Zhu

**Affiliations:** 1 State Key Laboratory of Rice Biology and Breeding, Ministry of Agriculture and Rural Affairs Key Laboratory of Molecular Biology of Crop Pathogens and Insect Pests, Institute of Insect Sciences, Zhejiang University, Hangzhou 310058, China; 2 Department of Plant Pests and Diseases, University of Brawijaya, Malang 65145, Indonesia; 3 Institute of Entomology, Northwest A&F University, Xianyang 712100, China; 4 Plant Protection Department, Faculty of Agriculture (Saba Basha), Alexandria University, Alexandria 21531, Egypt; 5 Shandong Lushou Seed Industry Co., Ltd., Shouguang 262700, China; 6 Hainan Institute, Zhejiang University, Sanya 572000, China

**Keywords:** biodiversity conservation, clothianidin, ecotoxicology, *Fejervarya cancrivora*, feeding behavior, locomotion, thyroid hormones

## Abstract

Clothianidin (CLO) is a widely used neonicotinoid insecticide in agricultural systems and may pose risks to non-target aquatic organisms, including amphibians. Here, we evaluated acute and sublethal effects of CLO on *Fejervarya cancrivora* tadpoles, an important predator of insect pests in rice paddy ecosystems. Acute toxicity tests (96 h) yielded an LC_50_ of 50.41 mg a.i./L (with LC_10_, LC_25_ and LC_30_ values of 15.35, 31.96 and 36.07 mg a.i./L, respectively). Sublethal exposure at these concentrations significantly reduced body weight, whole-body length, and hindlimb length during metamorphosis. CLO also altered thyroid hormone regulation, with T4 showing a dose-dependent increase, while T3 was elevated relative to controls but showed comparatively limited additional sensitivity to concentration and exposure duration. Locomotor activity was impaired under sublethal CLO exposure, reflected by reduced swimming distance and speed. In addition, frogs that developed from CLO-exposed tadpoles exhibited decreased feeding efficiency on brown planthoppers (*Nilaparvata lugens*) across developmental stages 46–48. Together, these findings demonstrate that CLO can affect amphibian development, endocrine regulation, and behavior at sublethal levels, highlighting the need to incorporate sublethal endpoints into ecological risk assessment and to promote pest management strategies that reduce impacts on biodiversity and ecosystem services.

## 1. Introduction

Neonicotinoids (NEOs) are a widely used class of systemic insecticides that have become integral to modern agricultural practices. Since their introduction in the 1990s, they have accounted for approximately 25% of global insecticide sales, with a broad spectrum of applications, ranging from cotton and maize to rice [1,2]. NEOs are highly effective in pest control, offering broad-spectrum activity and relatively low mammalian toxicity, making them a preferred choice in agriculture [3]. Among the most used NEOs in rice pest management is clothianidin, a second-generation compound. Like other NEOs, clothianidin (CLO) acts by mimicking acetylcholine and targeting nicotinic acetylcholine receptors (nAChRs) in insects, leading to overstimulation of the nervous system, paralysis, and death [2,4]. These insecticides are systemic, enabling uptake and translocation throughout plants [5]. However, plant uptake is often incomplete; depending on crop and application method, only a small fraction (reported to be up to ~28%) may be absorbed, while the remainder is deposited in soil and can be transported to aquatic environments [6]. This mechanism can also affect non-target organisms such as amphibians, given the conservation of cholinergic signaling pathways. Consistent with this concern, accumulating evidence indicates that neonicotinoid exposure can adversely affect amphibians, and recent syntheses emphasize growing ecological concern [7,8].

Despite their effectiveness, the widespread use of NEOs has raised significant ecological concerns. Due to their high-water solubility and environmental persistence, NEOs have been detected at harmful concentrations in soils and aquatic habitats worldwide [9,10]. NEOs have been reported in surface waters at ng/L levels and, in some settings, can reach higher ranges; a recent large-scale survey detected total concentrations of eight neonicotinoid insecticides from 2.3 to 1377.8 ng/L and reported sites exceeding chronic and acute toxicity thresholds [11]. These findings raise concerns about non-target effects on pollinators, birds, mammals, and amphibians [8,12,13,14].

The global consumption of pesticides has risen to approximately 3.5 million tons of active ingredients annually [15], while NEOs represent more than 25% of the global pesticide market/sales [16,17]. Residues have been detected frequently in fruits and vegetables, raising concerns about dietary exposure and potential health risks [14,18]. Their persistence and widespread use necessitate a closer examination of their impact on non-target organisms, particularly in agroecosystems. Regulatory bodies such as the European Union and the US Environmental Protection Agency have responded to these concerns over environmental risks by restricting or regulating certain NEOs, including imidacloprid and clothianidin [19,20,21]. However, NEOs are still widely used in agriculture, including through drone-based aerial spraying in rice cultivation [22,23]. This continued application raises concerns about potential impacts on non-target species in agricultural aquatic habitats (e.g., wetlands, rice paddies and associated surface waters) [11,14].

The brown planthopper (BPH, *Nilaparvata lugens* Stål) (Homoptera: Delphacidae) is a notorious and destructive pest of rice crops throughout Asia [24,25]. It damages rice plants by sucking phloem sap and laying eggs, and can also transmit harmful plant viruses, leading to significant yield losses [24,26]. Control of *N. lugens* relies heavily on insecticides, primarily nAChR competitive modulators such as NEOs [27]. However, due to the long-term, large-scale use of insecticides, *N. lugens* has evolved resistance to most insecticides used for control [28,29]. Recent syntheses further emphasize recurrent outbreaks and the need for sustainable BPH management strategies [30]. Disruption of the food web has led to increased BPH population densities, exacerbating damage to rice plantations and resulting in significant crop losses. Consequently, farmers have been compelled to use more pesticides, sometimes even creating their own mixtures [31].

Amphibians, such as the rice field frog (*Fejervarya cancrivora*), play a critical role in controlling pest populations in rice paddies by regulating insect populations, including BPH. In addition to pest control, amphibians provide valuable ecosystem services by consuming arthropod pests and also contribute provisioning services as a human food resource [32,33]. However, they are particularly vulnerable to pesticide exposure due to their permeable skin and life cycles that span both aquatic and terrestrial environments [34,35]. In rice agroecosystems, frogs are natural predators within paddy food webs, and pesticide applications may occur in the same habitats; therefore, pesticide exposure may reduce the ecological benefits that amphibians provide in pest regulation. The global decline of amphibians has been partly attributed to environmental pollution, which significantly affects their growth, survival, and behavior [36,37]. Additionally, the increasing use of pesticides in rice fields threatens amphibians’ roles in these ecosystems by disrupting food webs, shifting species composition, and leading to declines in certain species [38,39]. Exposure to pesticides also weakens their immune system, increasing their risk of infection [40]. Therefore, understanding how CLO affects the development of rice field frogs (*F. cancrivora*) and their ability to control BPH is critical for developing sustainable pest management strategies that balance effective crop protection with biodiversity conservation in rice ecosystems.

While previous studies have reported clothianidin’s impact on various organ systems of non-target organisms, the effects of long-term, low-dose exposure on amphibian physiology and behavior remain understudied [41,42,43]. Amphibians are regarded as ideal sentinel organisms due to their high sensitivity to pollutants and environmental stressors, making them effective indicators of ecosystem health [36,39]. Although the median lethal concentration (LC_50_) of NEOs to amphibian larvae is relatively high, sublethal exposures can induce a range of effects, including behavioral changes, delayed metamorphosis, and oxidative stress, which may contribute to population declines by reducing survival and predation efficiency [7,8,39]. This heightened sensitivity underscores the need for further investigation into how NEOs’ exposure affects amphibians’ ecological roles in agricultural settings, particularly in rice paddies, where these species are both abundant and vulnerable [32,33].

This study aims to investigate the effects of CLO on the development, behavior, and ecological role of *F. cancrivora* under controlled laboratory exposure conditions. We assessed CLO-related changes in growth, physiological processes, movement, and feeding performance. These results provide insight into the potential ecological implications of CLO exposure and may inform ecological risk assessment and pest management strategies that protect crop production and biodiversity in rice ecosystems.

## 2. Materials and Methods

### 2.1. Reagents and Chemicals

Clothianidin (purity 99.80%) was purchased from Toronto Research Chemical Co., Ltd. (Toronto, ON, Canada) (CAS No. 210880-92-5). A concentrated stock solution of CLO (40 mg/mL) was prepared in dimethyl sulfoxide (DMSO) (CAS No. 67-68-5), mixed until fully dissolved, and stored in the dark at 4 °C. Other chemicals, including ethyl acetate, n-hexane, acetonitrile, methanol, and acetone, were obtained from Shinopharma Chemical Reagents Co., Ltd. (Beijing, China). 3-Aminobenzocaine Ethyl Ester and Methanesulfonate (MS-222) were sourced from Sigma (St. Louis, MO, USA). All reagents used were of analytical grade.

### 2.2. Animal Husbandry

Tadpoles of *F. cancrivora* were obtained from the College of Agriculture and Biotechnology, Zhejiang University, Hangzhou, China. They were maintained in glass tanks (20 L water per tank) filled with carbon-filtered tap water at 17–19 °C under a 12 h light-dark cycle. Water quality parameters were controlled as follows: pH 7.0 ± 0.5, chlorine concentration 5 µg/L, and water hardness (CaCO_3_) approximately 150 mg/L. Tanks were continuously aerated using an air pump, and animals were inspected daily for general condition. During holding, water was renewed every 72 h to maintain water quality; salinity was not adjusted (freshwater conditions). Tadpoles were fed live Artemia three times daily. Developmental stages were determined according to the Gosner staging system [44]. Animal care and use were conducted following the Guide to the Management and Use of Laboratory Animals [45]. Unless stated otherwise, husbandry conditions were maintained throughout the study. Experimental exposures began at Gosner stage 26/27 for the acute toxicity, metamorphosis, thyroid hormone, and locomotor assays.

### 2.3. Acute Toxicity Test

An acute toxicity assay was conducted to determine the 96 h LC_50_ of CLO of *F. cancrivora* tadpoles [46]. Tadpoles were randomly assigned to exposure groups (four replicate tanks per concentration; 20 tadpoles per tank; 20 L water per tank). CLO exposure solutions were prepared from the 40 mg/mL DMSO. Nominal CLO concentrations of 0, 4, 11, 33, 100, and 300 mg/L were selected based on prior neonicotinoid toxicity studies in amphibians [47,48,49]. The 0 mg/L group served as the solvent control and received the same DMSO concentration as the highest-dose treatment (0.75% *v*/*v*). Mortality was recorded at 24, 48, 72, and 96 h; individuals were considered dead if they showed no movement in response to gentle stimulation. The 96 h LC_50_ (and LC_10_/LC_25_/LC_30_) values were estimated by probit analysis with 95% confidence intervals using SPSS (version 20.0; IBM Corporation, Armonk, NY, USA).

### 2.4. Metamorphosis Assay

Tadpoles were exposed to CLO to assess effects on metamorphosis. Exposure durations were set at 7, 14, and 21 days. Three sublethal concentrations were selected for subsequent assays: LC_10_ (15.35 mg a.i./L), LC_25_ (31.96 mg a.i./L), and LC_30_ (36.07 mg a.i./L), with a solvent control (0 mg/L CLO) that received the same DMSO concentration as the highest sublethal treatment (LC_30_; 0.09% *v*/*v* DMSO). For each exposure duration (7, 14, 21 d), four replicate tanks were used per treatment (20 tadpoles/tank; 20 L of water). At the end of each exposure period, 10 tadpoles were randomly sampled from each tank. Tadpoles from each tank were pooled to form a single biological replicate, resulting in four replicates per treatment group at each time point. Tadpoles were lightly anesthetized using 100 mg/L MS-222 prior to morphometric assessment. Body weight, whole-body length, and hindlimb length were recorded for each tadpole before tissue collection. Following measurement, whole-body tissue was collected under the same anesthetic conditions for subsequent hormone analysis.

### 2.5. Measurement of Thyroid Hormones

Systemic thyroid hormone levels in tadpoles were measured following the method described by Wang et al. [50]. Whole-body thyroid hormones (T4 and T3) were quantified using a commercially available ELISA kit (UScnlife, Wuhan, China), according to the manufacturer’s instructions. Whole-body extraction procedures, assay validation (parallelism and spike-recovery), recovery correction, and data reporting procedures are described in the Supplementary Information (Section S1). The detection limits for T4 and T3 were 45.5 pg/mL and 1.21 ng/mL, respectively. Hormones were measured in tadpoles from the control, LC_10_, LC_25_, and LC_30_ groups after 7, 14, and 21 days of exposure.

### 2.6. Measurement of Tadpole Locomotor Activity

Tadpole locomotor activity was quantified using a video-tracking system (Video-Track; ViewPoint Life Sciences, Montreal, QC, Canada), following Chen et al.; full protocol details are provided in Supplementary Information (Section S2) [51]. Briefly, tadpoles were tested individually on a 6-well plate (one tadpole per well) after a 10 min acclimation period. Locomotor endpoints were measured at 24, 48, 72, and 96 h in control and LC_10_, LC_25_, and LC_30_ groups (*n* = 6 per treatment per time point). The solvent control matched the DMSO concentration of the highest sublethal group (LC_30_).

### 2.7. Feeding Potential Assessment

Individuals at Gosner stage 46 (G46) were exposed to CLO and feeding performance was assessed repeatedly in the same individuals at Gosner stages G46, G47, and G48 after metamorphosis. Adults of BPH, a major rice pest, were used as prey. For each treatment, 4 frogs were tested. Each frog was placed individually in a cage containing 50 live BPH, and the number of BPH consumed was recorded after 24 h.

BPH were originally collected from rice fields at the Zijingang Campus of Zhejiang University, Hangzhou, China (30.3072° N, 120.0859° E). The colony was maintained for more than 30 generations on the susceptible rice variety Taichung Native 1 (TN1) under controlled insectary conditions (26 ± 1 °C, 70 ± 5% relative humidity, 16:8 h light/dark photoperiod).

### 2.8. Data Analysis

Statistical analyses were performed using SPSS v20.0 (IBM Corporation). Data normality and homogeneity of variance were assessed using the Kolmogorov–Smirnov and Levene’s tests, respectively. Results are presented as mean ± standard error of the mean (SEM). Treatment effects were evaluated using one-way ANOVA followed by Tukey’s post hoc test: morphometric endpoints and thyroid hormones were analyzed across treatments within each exposure duration (7, 14, 21 days), locomotor endpoints across treatments within each measurement time point (24, 48, 72, 96 h), and feeding (BPH consumed) across treatments within each developmental stage (G46, G47, G48). Developmental stage distributions were compared among treatments using a chi-square test. Stepwise regression was used to evaluate the relationships among predation (BPH consumed) and developmental stage (G), CLO concentration (X), and quadratic terms (G^2^, X^2^); non-significant interaction terms were excluded during model selection. Statistical significance was set at *p* < 0.05 (*) and *p* < 0.01 (**).

## 3. Results

### 3.1. Acute Toxicity of CLO to Tadpoles of F. cancrivora

The acute toxicity of CLO to *F. cancrivora* tadpoles was evaluated by exposing tadpoles (Gosner stages 26–27) to a graded concentration series for 96 h. Probit analysis yielded a 96 h LC_50_ of 50.41 mg a.i./L (95% CL: 44.60–57.42), with LC_10_, LC_25_, and LC_30_ values of 15.35 (8.38–21.16), 31.96 (26.42–37.44), and 36.07 mg a.i./L (30.64–41.72), respectively (Table 1). The concentration–response relationship showed an estimated slope of 0.037 ± 0.003, and model fit was acceptable (χ^2^ = 3.610, DF = 3). Control mortality remained < 10%, meeting the assay’s validity criteria. Mortality increased monotonically with CLO concentration, reaching 100% at 300 mg/L (Figure S1). The derived LC_10_, LC_25_, and LC_30_ values were used as the nominal sublethal exposure concentrations for subsequent assays.

### 3.2. Sublethal Effects of CLO on Metamorphosis and Performance-Related Endpoints in F. cancrivora Tadpoles

**Figure 1:**
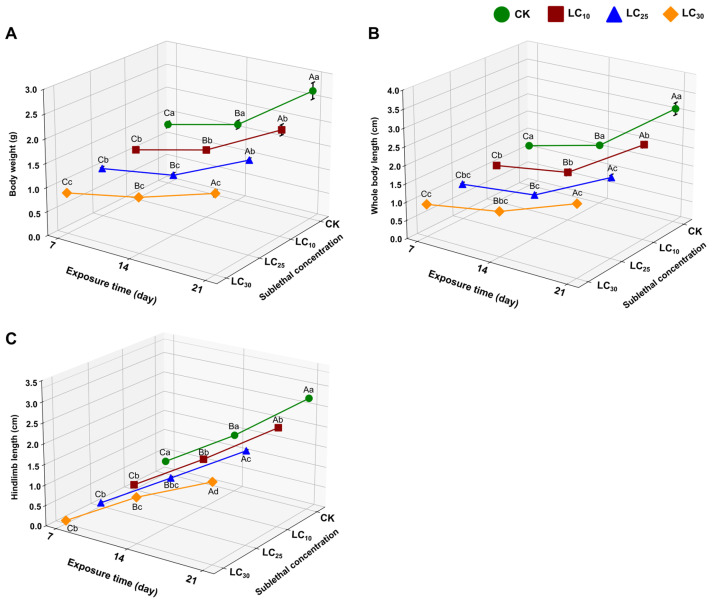
Effects of sublethal CLO exposure on morphometric development of *F. cancrivora* tadpoles. (**A**) Body weight, (**B**) whole-body length, and (**C**) hindlimb length after 7, 14, and 21 days of exposure to CK (solvent control), LC_10_ (15.35 mg a.i./L), LC_25_ (31.96 mg a.i./L), and LC_30_ (36.07 mg a.i./L). Bars represent mean ± SE. (*n* = 4 tanks per treatment per time point; 10 tadpoles measured per tank). Uppercase letters indicate significant differences among exposure durations within the same treatment; lowercase letters indicate significant differences among treatments within the same exposure duration (one-way ANOVA with Tukey’s post hoc test, *p* < 0.05).

Sublethal CLO exposure (LC_10_ = 15.35 mg/L, LC_25_ = 31.96 mg/L, LC_30_ = 36.07 mg/L) produced significant, time-dependent effects on tadpole growth and metamorphic development. Morphometric endpoints (body weight, whole-body length, hindlimb length) were quantified after 7, 14, and 21 days (Figure 1; Table S1), providing an integrated assessment of developmental progression under CLO exposure.

#### 3.2.1. Sublethal Effects of CLO on Body Weight, Body Length, and Hindlimb Length of *F. cancrivora*

The sublethal effects of CLO exposure on *F. cancrivora* tadpoles were assessed by measuring body weight, whole-body length, and hindlimb length (Figure 1; Table S1). Body weight differed significantly among treatments (F_3,36_ = 111.792, *p* < 0.001) and exposure durations (F_2,36_ = 524.521, *p* < 0.001), with a significant concentration × time interaction (F_6,36_ = 9.772, *p* < 0.001) (Figure 1A). Across all time points, body weight decreased with increasing CLO concentration, with the largest reductions observed in the LC_30_ group (36.07 mg/L) (Table S1).

A similar pattern was observed for whole-body length (Figure 1B; Table S1). Tadpoles exposed to CLO showed significantly reduced whole-body length relative to controls (F_3,36_ = 117.654, *p* < 0.001), and the magnitude of the effect increased over time (F_2,36_ = 1.147 × 10^3^, *p* < 0.001) (Figure 1B). For example, at 21 days, whole-body length decreased from 3.13 ± 0.14 cm (control) to 2.69 ± 0.07 cm (LC_10_), 2.17 ± 0.06 cm (LC_25_), and 1.96 ± 0.13 cm (LC_30_) (Table S1).

**Figure 2:**
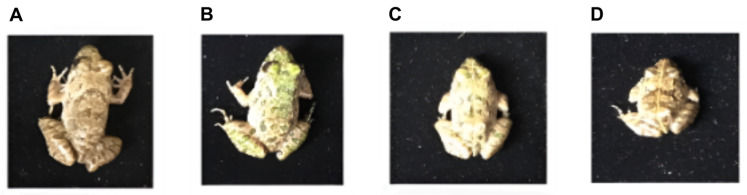
Representative developmental phenotypes of *F. cancrivora* tadpoles after 7 days of CLO exposure. (**A**) CK (solvent control), (**B**) LC_10_ (15.35 mg a.i./L), (**C**) LC_25_ (31.96 mg a.i./L), and (**D**) LC_30_ (36.07 mg a.i./L).

Hindlimb length, a key marker of metamorphic progression, also declined significantly under CLO exposure (Figure 1C; Table S1). At 21 days, hindlimb length decreased from 2.75 ± 0.03 cm (control) to 2.41 ± 0.03 cm (LC_10_), 2.25 ± 0.01 cm (LC_25_), and 1.94 ± 0.04 cm (LC_30_), indicating impaired limb development at higher sublethal concentrations (Table S1). Representative developmental phenotypes at day 7 are shown in Figure 2. Consistent with the morphometric data (Table S1; Figure 1), photographed individuals in the CLO-exposed groups appeared smaller than controls, with the greatest reduction in size observed at LC_25_ and LC_30_ (Figure 2).

#### 3.2.2. Thyroid Hormone Level

**Figure 3:**
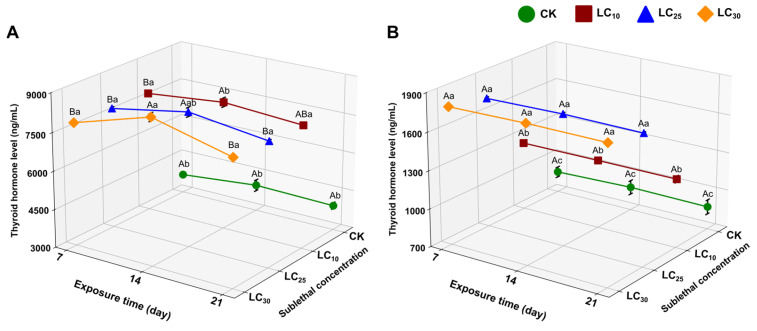
Effects of sublethal CLO exposure on thyroid hormone levels in *F. cancrivora* tadpoles. (**A**) T3 and (**B**) T4 levels after 7, 14, and 21 days of exposure to CK (solvent control), LC_10_ (15.35 mg a.i./L), LC_25_ (31.96 mg a.i./L), and LC_30_ (36.07 mg a.i./L). Bars represent mean ± SE (*n* = 4). Uppercase letters indicate significant differences among exposure durations within the same treatment, and lowercase letters indicate significant differences among treatments within the same exposure duration (one-way ANOVA followed by Tukey’s post hoc test, *p* < 0.05).

The effects of CLO on thyroid hormone levels were evaluated by measuring total body T3 and T4 levels in *F. cancrivora* tadpoles following exposure to LC_10_ (15.35 mg a.i./L), LC_25_ (31.96 mg a.i./L), and LC_30_ (36.07 mg a.i./L) for 7, 14, and 21 days (Figure 3; Table S2). Relative to the control (CK), T3 levels were significantly elevated in all CLO treatments at each exposure duration (Figure 3A; Table S2). At 7 and 21 days, T3 did not differ among LC_10_–LC_30_, whereas on 14 days, T3 was highest at LC_30_ (Table S2). In contrast, T4 showed a clear concentration-dependent increase across all time points, with LC_25_ and LC_30_ consistently higher than CK and LC_10_ (Table S2; Figure 3B). Across the 7–21 days exposure window, T4 values were broadly stable within each concentration group, indicating that treatment-related differences were driven primarily by CLO concentration rather than exposure duration (Table S2). Together, these results indicate that CLO exposure markedly increases thyroid hormone levels, with T4 exhibiting the strongest concentration-related response.

#### 3.2.3. Effect on Locomotor Behavior

**Figure 4:**
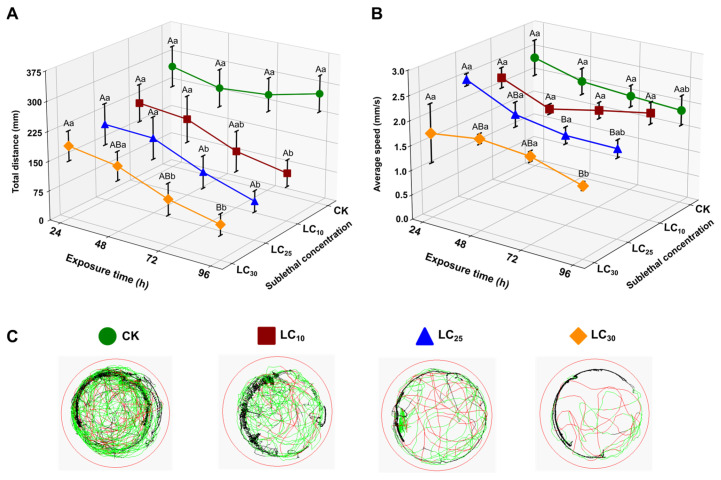
Effects of sublethal CLO exposure on locomotor behavior of *F. cancrivora* tadpoles. (**A**) Total distance traveled (mm), (**B**) average speed (mm/s), and (**C**) representative movement tracks after 24, 48, 72, and 96 h of exposure to CK, LC_10_, LC_25_, and LC_30_. Track color indicates instantaneous speed (black < 4 mm/s, green 4–20 mm/s, red > 20 mm/s). Bars represent mean ± SE (*n* = 6). Uppercase letters indicate significant differences among exposure times within the same treatment, and lowercase letters indicate significant differences among treatments within the same exposure time (one-way ANOVA with Tukey’s post hoc test, *p* < 0.05).

The impact of CLO concentration and exposure duration on locomotion was assessed by measuring total distance traveled and average swimming speed (Figure 4; Table S3). Across time points, locomotor performance declined with increasing CLO concentration, with the clearest suppression observed at 96 h. At 96 h, total distance traveled decreased from 282.62 ± 30.94 in the control to 122.55 ± 22.81 (LC_10_), 97.58 ± 17.76 (LC_25_), and 87.78 ± 17.76 mm (LC_30_) (*p* < 0.05, Table S3; Figure 4A). At earlier time points (24–48 h), distance did not differ significantly among treatments (Table S3).

Average speed showed a more modest treatment effect (Figure 4B; Table S3). At 96 h, LC_30_ exhibited a significantly lower speed (1.44 ± 0.05) than LC_10_ (2.17 ± 0.15), while the control and LC_25_ groups were intermediate (Table S3). Movement tracks further supported these patterns: control tadpoles displayed broader spatial exploration, whereas CLO-exposed tadpoles showed more restricted trajectories and reduced spatial coverage, particularly at higher concentrations (Figure 4C).

#### 3.2.4. Effect on Feeding Behavior

**Figure 5:**
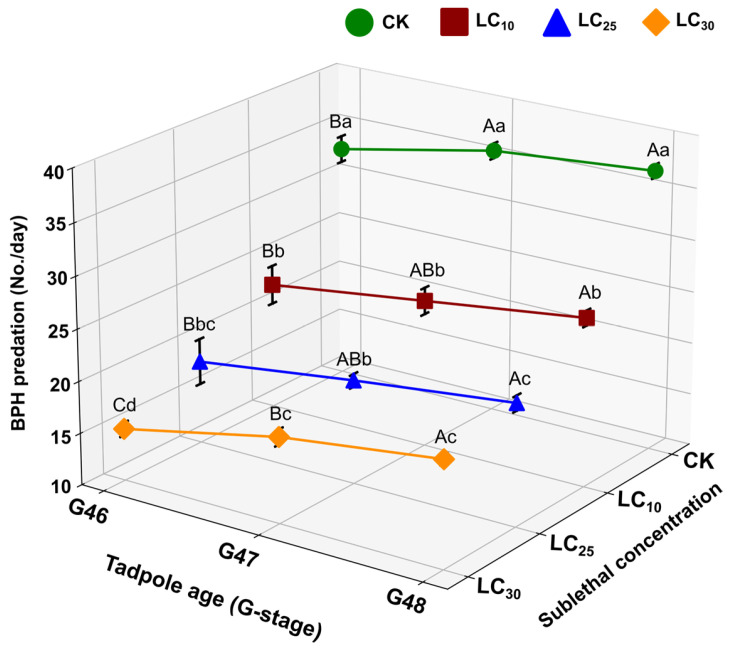
Effects of sublethal CLO exposure on predation of BPH by *F. cancrivora* across Gosner stages G46–G48. The number of adult BPH consumed over 24 h by frogs developed from CLO-exposed tadpoles. Bars represent mean ± SE (*n* = 4 frogs per treatment per stage). Uppercase letters indicate significant differences among developmental stages within a given insecticide concentration; lowercase letters indicate significant differences among insecticide concentrations within a given developmental stage (one-way ANOVA with Tukey’s post hoc test, *p* < 0.05).

The feeding potential of frogs that developed from CLO-exposed tadpoles was assessed by measuring the quantifying predation on adult BPH over 24 h at Gosner stages G46, G47, and G48 (Figure 5; Table S4). Across all stages, BPH consumption declined significantly with increasing CLO concentration (Table S4). For example, at G46, mean consumption decreased from 32.75 ± 0.85 in control to 22.25 ± 1.25 (LC_10_), 18.00 ± 1.47 (LC_25_), and 15.00 ± 0.40 (LC_30_) (Table S4). Similar concentration-related reductions were observed at G47 and G48, with the lowest consumption consistently recorded in the LC_30_ group (Table S4).

**Figure 6:**
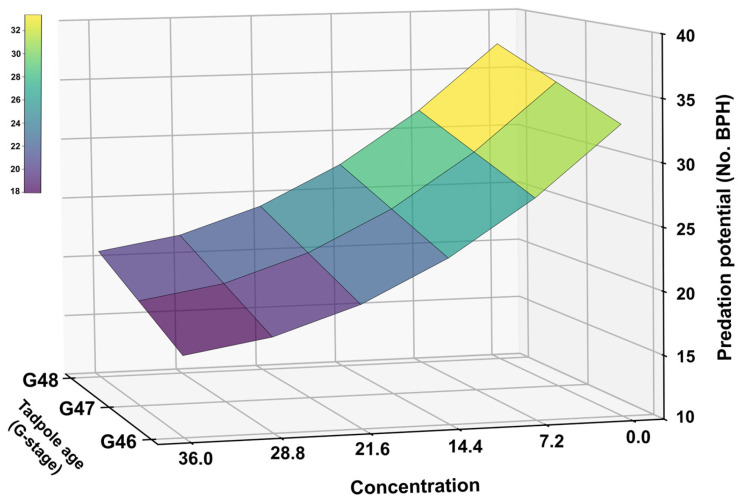
Response surface illustrating the combined effects of developmental stage and CLO concentration on BPH predation by *F. cancrivora*. The surface is based on the fitted regression model relating predation (BPH consumed per 24 h) to developmental stage (G46–G48) and CLO concentration.

Regression analysis further supported a negative association between CLO concentration and feeding performance across developmental stages (Figure 6). The final model (Predation = −66.2917 + 2.15625G − 0.8421X + 0.0106X^2^) was highly significant (F_3,47_ = 323.8478, *p* = 1 × 10^−7^) and explained a large proportion of variation in predation (adjusted R^2^ = 0.9537). Consistent with the response surface, predicted BPH consumption increased with developmental stage (G46–G48) but declined with increasing CLO concentration, with a stronger reduction at higher concentrations (Figure 6).

## 4. Discussion

Environmental contamination by neonicotinoid insecticides, such as CLO, represents an important chemical stressor for non-target wildlife, including amphibians, due to their permeable skin, biphasic life cycle, and multiple exposure pathways [47,52]. Although amphibians are not the primary targets of NEOs, evidence indicates that both acute toxicity and sublethal physiological disruptions can occur, affecting growth, behavior, endocrine regulation, and ultimately survival [53,54,55,56]. This study provides a comprehensive assessment of the toxicological effects of CLO on *F. cancrivora* tadpoles, revealing significant impacts on survival, metamorphosis, thyroid hormone regulation, locomotion, and predatory behavior.

The acute toxicity assessment of CLO on *F. cancrivora* tadpoles revealed a 96 h LC_50_ of 50.41 mg a.i./L. In addition to the LC_50_, lower concentrations were determined, with the LC_10_, LC_25_, and LC_30_ values calculated at 15.35 mg/L, 31.96 mg/L, and 36.07 mg/L, respectively. These values align with moderate acute toxicity observed in other amphibian species exposed to NEOs, such as imidacloprid, which reported LC_50_ values ranging from 52.6 mg/L to 366 mg/L [47,48,49]. Notably, *F. cancrivora* appears to have a higher sensitivity to CLO than other amphibian species, such as *Silurana tropicalis*, whose LC_50_ for CLO exceeded 100 mg/L [57]. Species-specific differences in toxicity may arise from differences in physiology, metabolic detoxification pathways, developmental rates, and experimental conditions.

While the LC_50_ provides a valuable benchmark, it is crucial to emphasize that focusing solely on lethal concentrations does not fully capture the ecological risks posed by CLO exposure. The LC_10_ (15.35 mg/L) and LC_25_ (31.96 mg/L) values are informative because they correspond to 10% and 25% mortality, respectively. In fluctuating amphibian populations, even at lower concentrations, such mortality rates may still have significant ecological impacts. Sublethal effects from NEOs, such as delayed development, growth inhibition, and behavioral changes, have increasingly been recognized as critical threats to amphibians [58]. The actual environmental risk of pesticide exposure often arises from chronic, sublethal effects that accumulate over time rather than from sudden mass mortality. Consequently, the concentrations derived from our acute toxicity tests (LC_10_, LC_25_, and LC_30_) serve as an essential threshold for investigating sublethal impacts that may contribute to population declines. Global amphibian decline underscores the necessity of incorporating sublethal endpoints into risk assessment models to more accurately assess ecological consequences [59].

Sublethal exposure to CLO significantly impaired the growth and development of *F. cancrivora* tadpoles, as evidenced by dose- and time-dependent reductions in body weight, body length, and hindlimb length. The inhibition of hindlimb development, a primary indicator of metamorphic progress, is particularly concerning. As hindlimbs are crucial for locomotion and survival in the terrestrial environment, this disruption could compromise post-metamorphic fitness [60]. The observed developmental delays align with previous research showing that growth and metamorphosis can be disrupted in amphibians exposed to NEOs [61,62]. Specifically, Jenkins et al. documented disrupted growth and delayed metamorphosis in *Xenopus laevis* tadpoles exposed to CLO and thiamethoxam [61], while Brodeur and Fonseca Peña reported similar metamorphic delays in various amphibian species exposed to thiamethoxam and imidacloprid [62].

The growth inhibition observed in this study suggests that CLO exposure may interfere with fundamental physiological processes, including energy metabolism, nutrient assimilation, and endocrine regulation. Amphibian metamorphosis is an energetically demanding and highly synchronized process that transitions tadpoles from an aquatic larval form to a terrestrial or semi-aquatic adult. Environmental stressors, such as pesticide exposure, can disrupt this process, resulting in delayed or incomplete development. Delayed metamorphosis has been shown to increase susceptibility to predation, desiccation, and environmental stress, thereby reducing both survival and reproductive fitness [63,64,65]. These developmental delays can have cascading ecological consequences, especially in habitats such as rice paddies, where *F. cancrivora* plays an essential role in pest control. The sublethal effects of CLO could disrupt population dynamics, potentially leading to declines in amphibian numbers, with implications for local biodiversity and ecosystem function [1,23]. Furthermore, a smaller size at metamorphosis, a direct consequence of developmental delays, has been linked to reduced overwintering survival, delayed sexual maturity, increased susceptibility to predation, and overall lower reproductive success in amphibians [60]. Even if tadpoles survive CLO exposure and complete metamorphosis, they may enter the terrestrial environment as compromised individuals, with reduced fitness and a diminished ability to contribute to future generations.

Amphibian metamorphosis is primarily regulated by thyroid hormones (T3 and T4), which act through the hypothalamic-pituitary-thyroid (HPT) axis to orchestrate the dramatic cellular and morphological changes during development [66,67]. In this study, CLO exposure led to significant increases in both T3 and T4 levels across all treatment groups, with T4 showing a dose-dependent elevation. Despite elevated thyroid hormone levels, tadpoles exhibited stunted growth and delayed hindlimb development, indicating a disruption of thyroid hormone signaling at the receptor or tissue level. The uncoupling of elevated thyroid hormone levels from expected physiological outcomes suggests that CLO may interfere with thyroid hormone action, potentially inducing thyroid hormone resistance. While high concentrations of thyroid hormones typically accelerate metamorphosis, our findings suggest that CLO exposure might block the appropriate physiological response, potentially by interfering with thyroid hormone receptors (TRs), disrupting the transport of THs into cells, or altering downstream signaling pathways [68]. This phenomenon of thyroid resistance has been observed with other NEOs, suggesting a complex mode of endocrine disruption [69].

Interestingly, the elevation in T4 levels could result from multiple mechanisms, including stimulation of the thyroid gland to overproduce T4, disruption of the feedback loop that halts typical TH synthesis, or inhibition of T4 clearance [70]. Furthermore, the increase in T3 levels may reflect enhanced peripheral conversion of T4 to T3 by deiodinase enzymes. Despite these elevated hormone levels, the absence of normal metamorphic acceleration strongly suggests that CLO interferes with key aspects of thyroid hormone regulation and action, thereby impairing tadpole development. These findings underscore the complexity of endocrine disruption caused by CLO and highlight the need for further investigation into how neonicotinoids affect thyroid hormone signaling in amphibians. Interference with thyroid hormone signaling not only delays metamorphosis but may also have long-term consequences for amphibian survival and reproductive success.

Behavior is a sensitive and integrative indicator of overall health and neurological integrity. In this study, sublethal exposure to CLO significantly impaired the locomotor behavior of *F. cancrivora* tadpoles, as evidenced by reduced total distance traveled and lower swimming speed, with hypoactivity increasing in a dose- and time-dependent manner. Movement path analysis further showed that CLO-exposed tadpoles covered smaller areas and exhibited more restricted exploratory behavior than the active, wide-ranging control group. These impairments in mobility are indicative of neurotoxic effects, likely mediated through the interference with vertebrate nicotinic acetylcholine receptors (nAChRs), despite their lower affinity for neonicotinoids compared to insect nAChRs [59,71,72].

NEOs, such as CLO, act as agonists of nAChRs, overstimulating and potentially paralyzing insect species. While vertebrate nAChRs exhibit lower sensitivity to these chemicals, they are not immune to their effects, especially at concentrations typically found in contaminated environments. The observed lethargy and reduced activity in CLO-exposed tadpoles are hallmark signs of neurological impairment in aquatic vertebrates exposed to neurotoxic pesticides. Supporting this, Shinya et al. demonstrated that neonicotinoids can alter brain catecholamine levels in frogs, providing a neurochemical basis for behavioral changes [59]. Additionally, research by Woodley et al. has shown that chronic exposure to mixtures of neonicotinoids and other pesticides can physically alter the brain structure of larval amphibians, suggesting a morphological underpinning for long-term behavioral deficits [71]. Similarly, sulfoxaflor, another neonicotinoid compound, has been shown to cause growth disturbances and inflammatory responses in tadpoles, highlighting a shared neurotoxic pathway among insecticides [72]. The ecological implications of these behavioral impairments are significant. Reduced mobility diminishes tadpoles’ ability to forage efficiently, potentially exacerbating the observed growth inhibition. More critically, the increased lethargy and hypoactivity render tadpoles more susceptible to predation. This dual effect, impairing foraging capacity and heightened predation risk, places CLO-exposed tadpoles at a considerable survival disadvantage within their natural ecosystem, potentially leading to reduced population sustainability.

This study demonstrates that CLO exposure during the larval stage has lasting effects on adult *F. cancrivora* feeding behavior, with significant reductions in predation on BPH that persist in adulthood in a dose-dependent manner. Regression analysis revealed that CLO exposure accounted for more than 95% of the variance in predatory performance, underscoring the enduring impact of larval pesticide exposure on adult ecological function. In rice paddy ecosystems, *F. cancrivora* functions as a natural pest-control agent, but CLO exposure impairs its ability to control BPH, thereby creating an ecological feedback loop. The use of pesticides to manage pests inadvertently reduces the effectiveness of frogs as predators, potentially increasing reliance on chemical control and perpetuating environmental degradation [47,73,74]. The reduced feeding ability of CLO-exposed frogs may result from neurological damage, developmental abnormalities, or general health decline. Regardless of the exact cause, the result is a less effective predator, disrupting local food webs and diminishing the resilience of agroecosystems. This loss of function, combined with threats to frog survival and reproduction, poses significant risks to amphibian populations in agricultural landscapes.

This study shows that CLO exposure disrupts *F. cancrivora* by causing endocrine disruption, growth inhibition, neurotoxicity, and behavioral impairment, with lasting effects on adult predation. Even at the sublethal concentrations tested, CLO significantly affected individual fitness and ecosystem functions. These results highlight that acute toxicity metrics (LC_50_) are insufficient for assessing ecological risks, and regulatory frameworks must include sublethal endpoints like growth, endocrine function, and behavior [7,75,76]. Given amphibians’ vulnerability to chemical pollution, more precautionary approaches in pesticide regulation are essential to protect these species and the services they provide [77].

While our experiments were conducted under controlled laboratory conditions using nominal exposure concentrations, future studies could build on these findings by verifying CLO residues in water and/or tissues and including mechanistic endpoints (e.g., thyroid-axis gene expression and histopathology), ideally using more realistic pesticide mixtures and larger-scale tank or outdoor studies.

## 5. Conclusions

This study shows that clothianidin (CLO) can adversely affect *Fejervarya cancrivora* across multiple biological levels, including growth, thyroid hormone regulation, locomotor performance, and feeding capacity. Sublethal exposure (LC_10_, LC_25_ and LC_30_) consistently reduced body weight, whole-body length, and hindlimb length during metamorphosis, altered thyroid hormone profiles, particularly by increasing T4 in a dose-dependent manner (with T3 also elevated relative to controls), and impaired swimming activity. Importantly, frogs that developed from CLO-exposed tadpoles exhibited reduced predation on brown planthoppers (*Nilaparvata lugens*), indicating that CLO exposure may weaken the pest-control function provided by this species in rice paddy ecosystems. These findings support the broader view that protecting amphibian populations can help sustain natural pest suppression as part of biodiversity-friendly integrated pest management, rather than increasing reliance on chemical control alone. Collectively, these findings illustrate that reliance on acute lethality metrics alone (e.g., LC_50_) may underestimate ecological risk. Incorporating sublethal and longer-term endpoints, such as development, endocrine disruption, behavior, and functional performance, into pesticide risk assessment would improve protection of amphibians and the ecosystem services they support.

## Figures and Tables

**Table 1:** Acute toxicity of CLO to *F. cancrivora* tadpoles after 96 h exposure.

Tadpoles Stage	LC mg a.i./L (95% CL)				Slope ± SE	X^2^ (DF)
	LC_10_	LC_25_	LC_30_	LC_50_		
G26/27	15.35 (8.38–21.16)	31.96 (26.42–37.44)	36.07 (30.64–41.72)	50.41 (44.60–57.42)	0.037 ± 0.003	3.610 (3)

LC_10_, LC_25_, LC_30_, and LC_50_ represent the CLO concentrations (mg a.i./L) causing 10%, 25%, 30%, and 50% mortality, respectively. Values are presented with 95% confidence limits (CL). X^2^ indicates the goodness of fit for the probit model, with degrees of freedom (DF) in parentheses.

## Data Availability

The original contributions presented in this study are included in the article/Supplementary Materials. Further inquiries can be directed to the corresponding author.

## Supplementary Materials

The following supporting information can be downloaded at: https://www.mdpi.com/article/10.3390/toxics14030243/s1, Figure S1: Concentration-response curve for acute toxicity of CLO to *F. cancrivora* tadpoles following 96-h exposure. Mortality (%) is plotted against nominal CLO concentrations (mg/L). The 96 h LC_50_ was estimated using probit analysis to be 50.41 mg/L (95% CI: 44.60–57.42 mg/L). Data points represent mean ± SE (*n* = 4 replicate tanks per concentration; 20 tadpoles per tank). The dashed vertical line indicates the LC_50_ estimate; Figure S2: Representative tadpole movement trajectories recorded using the Video-Track system (ViewPoint Life Sciences, Montreal, Canada) following CLO exposure for 24, 48, 72, and 96 h. Trajectories are shown for (A) 24 h, (B) 48 h, (C) 72 h, and (D) 96 h. Line color indicates instantaneous speed: black (<4 mm/s), green (4–20 mm/s), and red (>20 mm/s); Table S1: Effects of sublethal CLO exposure on growth and metamorphic development of *F. cancrivora* tadpoles; Table S2: Effects of sublethal CLO exposure on thyroid hormone levels (T3 and T4) in *F. cancrivora* tadpoles; Table S3: Locomotor performance of *F. cancrivora* tadpoles following CLO exposure (CK, LC_10_, LC_25_, LC_30_) was measured at 24, 48, 72, and 96 h using the Video-Track system; Table S4: Effects of CLO exposure on predation of BPH by *F. cancrivora* across developmental stages (G46–G48).
