# Supplementary material for: Sublethal Clothianidin Exposure Impairs Development, Thyroid Hormones, Locomotion and Predation in Fejervarya cancrivora from Rice Paddy Ecosystems
Source: Toxics. 2026 Mar 11;14(3):243. doi: 10.3390/toxics14030243 (PMC13030093; doi:10.3390/toxics14030243)
Supplement: Supplementary file 1 [file toxics-14-00243-s001.zip › toxics-4166011-supplementary.pdf]

Supplementary material

Sublethal Clothianidin Exposure Impairs Development, Thyroid Hormones, Locomotion and Predation in *Fejervarya cancrivora* from Rice Paddy Ecosystems

Joko Pilianto [1,2], Amr Abou El-Ela [1,3,4], Asim Munawar [1], Xiangfen Zhang [1], Dun Wang [5], Abid Ali Soomro [1], Naved A. Ansari [1], Wenwu Zhou [1], and Zengrong Zhu [1,6*]

1 State Key Laboratory of Rice Biology and Breeding, Ministry of Agriculture and Rural Affairs Key laboratory of Molecular Biology of Crop Pathogens and Insect Pests, Institute of Insect Sciences, Zhejiang University, Hangzhou 310058, China; Joko.pilianto@gmail.com & asim_munawar@zju.edu.cn & 22316277@zju.edu.cn & abid_ali26@yahoo.com & navedahmad.ansari@gmail.com & wenwuzhou@zju.edu.cn & zrzhu@zju.edu.cn.

2 Department of Plant Pests and Diseases, University of Brawijaya, Malang 65145, Indonesia.

3 Plant Protection Department, Faculty of Agriculture (Saba Basha), Alexandria University, Alexandria 21531, Egypt; dr_amraboelela@alexu.edu.eg.

4 Shandong Lushou Seed Industry Co., LTD., Shouguang 262700, China.

5 Institute of Entomology, Northwest A&F University, Xianyang 712100, China; wanghande@nwsuaf.edu.cn.

6 Hainan Institute, Zhejiang University, Sanya 572000, China.

* Correspondence: zrzhu@zju.edu.cn.

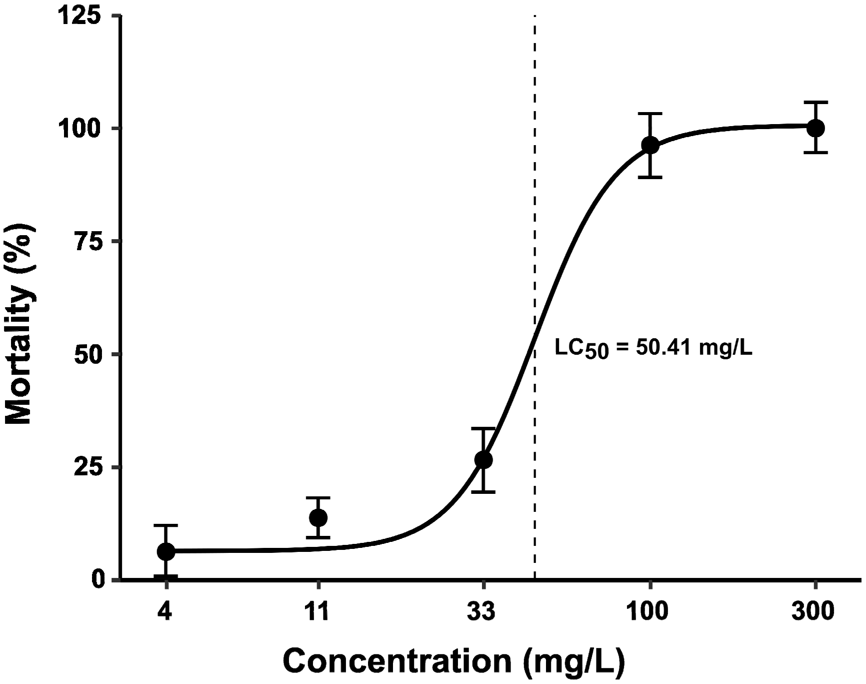

Figure S1. Concentration-response curve for acute toxicity of CLO to *F. cancrivora* tadpoles following 96-hour exposure.

Mortality (%) is plotted against nominal CLO concentrations (mg/L). The 96-h LC_{50} was estimated using probit analysis to be 50.41 mg/L (95% CI: 44.60–57.42 mg/L). Data points represent mean ± SE (n = 4 replicate tanks per concentration; 20 tadpoles per tank). The dashed vertical line indicates the LC_{50} estimate.

Section S1. Thyroid hormone extraction and assay

Thyroid hormone extraction from whole-body *F. cancrivora* tadpoles was performed following the method described by Chen et al. (2012) and Wang et al. (2013) [1, 2]. Briefly, 10 tadpoles from each tank were homogenized in methanol: ammonia (99:1, v/v) containing 6-n-propyl-2-thiouracil. The homogenates were centrifuged at 6,000 × g for 15 min at 4 °C, and supernatants were evaporated to dryness. The residues were reconstituted in a barbital buffer and centrifuged again; the resulting aqueous phase was used for ELISA. Assay validity was confirmed through parallelism and spike-recovery testing. Mean recovery rates were 63.7 ± 0.8% for T3 and 72.7 ± 0.7% for T4. Hormone concentrations were corrected for recovery and are reported as ng/mL (ELISA readout).

Section S2. Tadpole locomotion activity assay

Locomotor activity of *F. cancrivora* tadpoles was assessed using the Video-Track system (ViewPoint Life Sciences, Montreal, Canada), following protocols described by Chen et al. (2012). Tadpoles were placed individually into wells of a 6-well plate (one tadpole per well). After a 10 min acclimation period, swimming behavior was recorded under a 40 min dark-to-light cycle, consisting of

two alternating 10 min periods of visible light and 10 min of infrared darkness. Data on movement frequency, total distance traveled, and movement duration were recorded every 60 s [2]. Each treatment and time point was assessed using one 6-well plate (n = 6 tadpoles per treatment per time point). Data were exported from Video-Track and summarized in OpenOffice.Org 2.4.

A. 24 hours

	R1	R2	R3	R4	R5	R6
CK	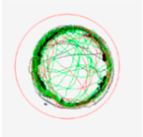	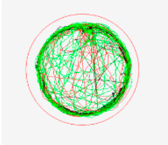	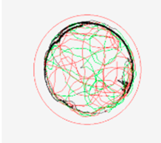	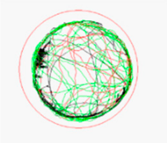	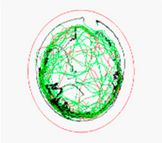	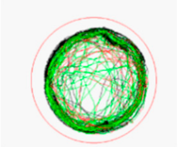
LC_{10}	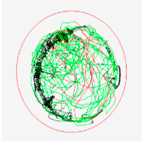	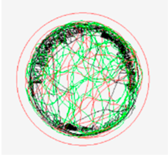	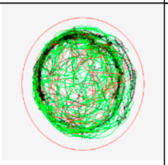	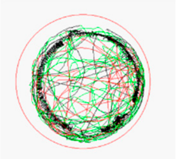	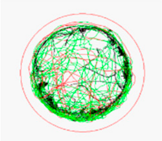	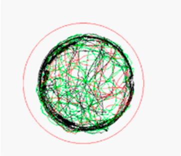
LC_{25}	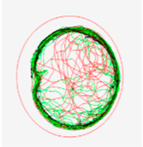	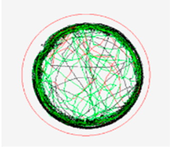	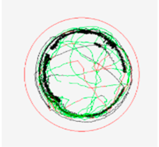	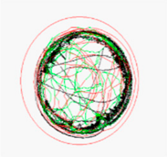	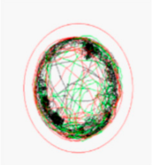	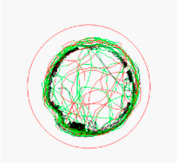
LC_{30}	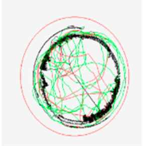	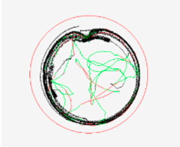	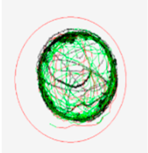	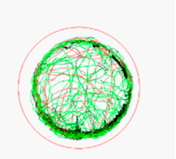	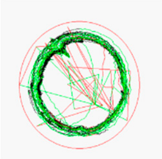	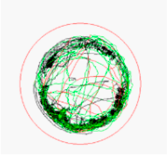

B. 48 hours

	R1	R2	R3	R4	R5	R6
CK	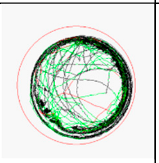	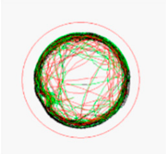	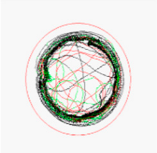	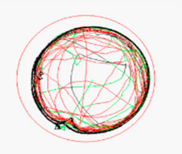	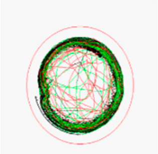	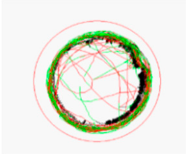

LC10	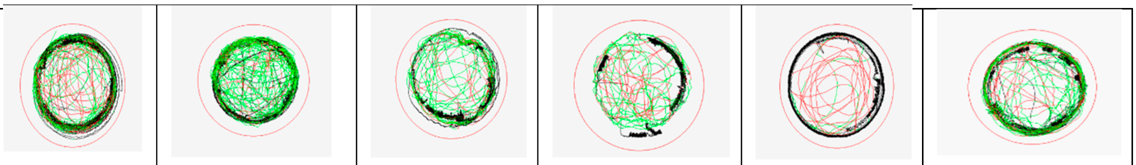					
LC25	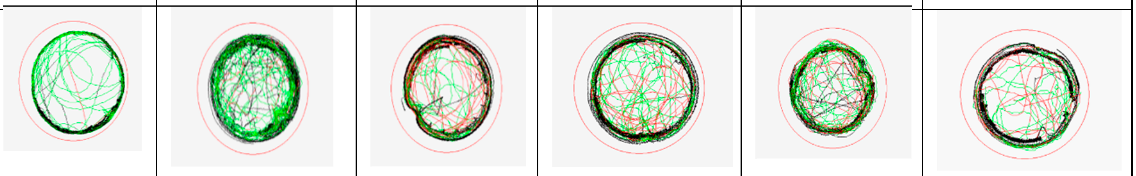					
LC30	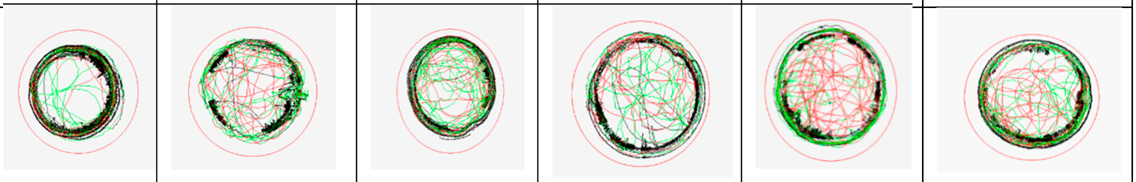					

C. 72 Hours

	R1	R2	R3	R4	R5	R6
CK	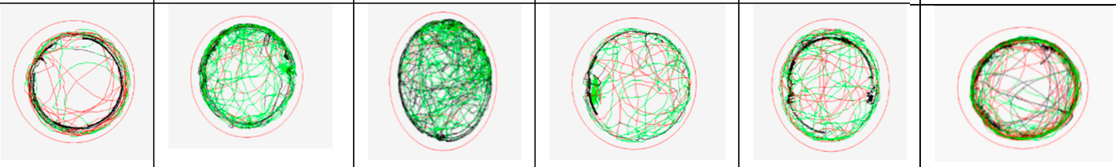					
LC10	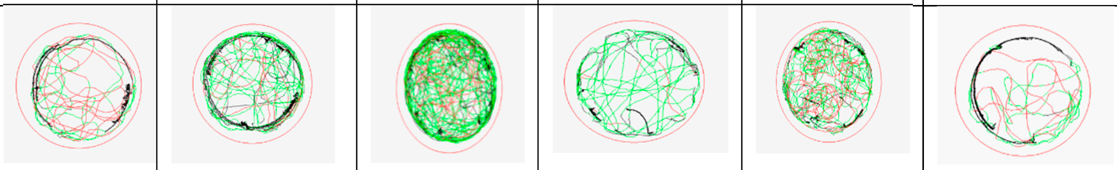					
LC25	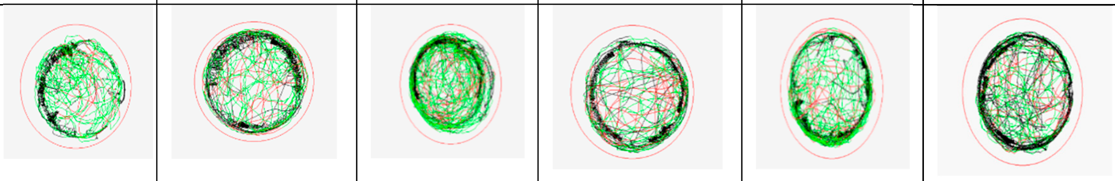					
LC30	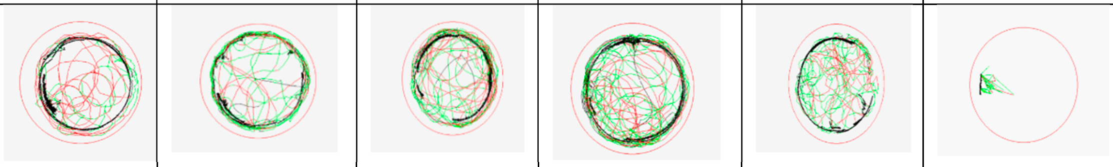					

D. 96 Hours

	R1	R2	R3	R4	R5	R6

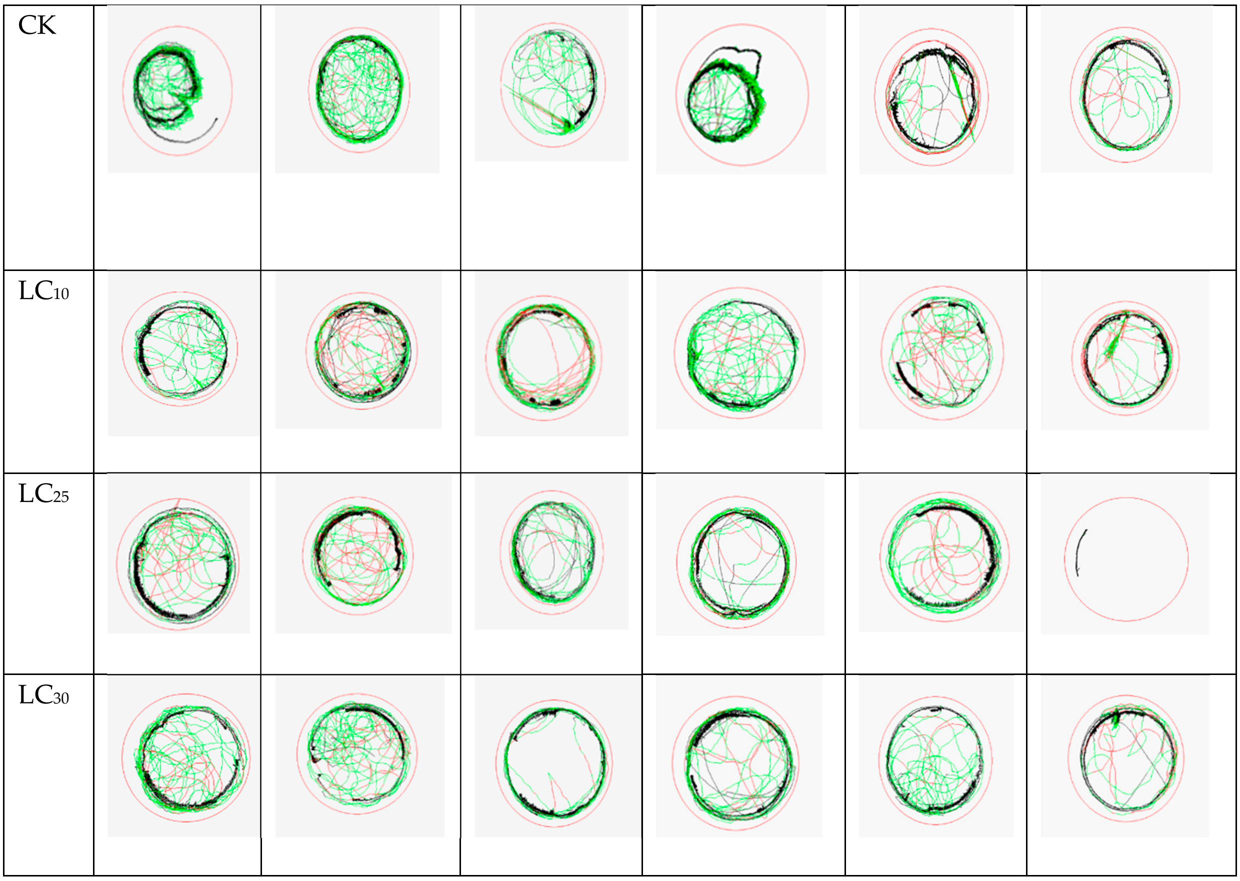

Figure S2. Representative tadpole movement trajectories recorded using the Video-Track system (ViewPoint Life Sciences, Montreal, Canada) following CLO exposure for 24, 48, 72, and 96 h. Trajectories are shown for **(A)** 24 h, **(B)** 48 h, **(C)** 72 h, and **(D)** 96 h. Line color indicates instantaneous speed: black (< 4 mm/sec), green (4–20 mm/sec), and red (> 20 mm/sec).

Table S1. Effects of sublethal CLO exposure on growth and metamorphic development of *F. cancrivora* tadpoles.

**Measurement**	**Concentration**	**Time (Days)**		
		**7**	**14**	**21**
Body weight (g)	CK	1.31 ± 0.08^{a}	1.65 ± 0.14^{a}	2.69 ± 0.15^{a}
	LC_{10}	1.08 ± 0.02^{b}	1.44 ± 0.03^{b}	2.21 ± 0.05^{b}
	LC_{25}	1.01 ± 0.03^{b}	1.25 ± 0.06^{c}	1.93 ± 0.03^{b}
	LC_{30}	0.85 ± 0.02^{c}	1.15 ± 0.03^{c}	1.62 ± 0.06^{c}
Whole body length (cm)	CK	1.17 ± 0.04^{a}	1.66 ± 0.04^{a}	3.13 ± 0.14^{a}
	LC_{10}	1.04 ± 0.04^{b}	1.34 ± 0.03^{b}	2.69 ± 0.07^{b}
	LC_{25}	0.97 ± 0.03^{c}	1.19 ± 0.03^{c}	2.17 ± 0.06^{c}
	LC_{30}	0.89 ± 0.05^{c}	1.23 ± 0.05^{bc}	1.96 ± 0.13^{c}
Hindlimb length (cm)	CK	0.33 ± 0.04^{a}	1.43 ± 0.06^{a}	2.75 ± 0.03^{a}
	LC_{10}	0.12 ± 0.01^{b}	1.22 ± 0.02^{b}	2.41 ± 0.03^{b}
	LC_{25}	0.09 ± 0.01^{b}	1.16 ± 0.02^{bc}	2.25 ± 0.01^{c}
	LC_{30}	0.08 ± 0.02^{b}	1.12 ± 0.02^{c}	1.94 ± 0.04^{d}

Body weight (g), whole-body length (cm), and hindlimb length (cm) were measured after 7, 14, and 21 days of exposure to the solvent control (CK) or CLO at LC_{10}, LC_{25}, and LC_{30} (15.35, 31.96, and 36.07 mg a.i./L, respectively). Values are presented as mean ± SE (*n* = 4 tanks per treatment per time point; 10 tadpoles measured per tank).

Different lowercase letters within the same measurement and time point indicate significant differences among treatments (one-way ANOVA with Tukey's post hoc test, $p < 0.05$). CK = solvent control.

Table S2. Effects of sublethal CLO exposure on thyroid hormone levels (T3 and T4) in *F. cancrivora* tadpoles.

**Measurement**	**Concentration**	**Time (Days)**		
		**7**	**14**	**21**
T3	CK	3737.00 ± 29.01^{b}	4039.50 ± 148.40^{b}	3969.50 ± 76.63^{b}
	LC_{10}	7737.00 ± 44.82^{a}	8034.50 ± 114.99^{b}	7814.50 ± 30.10^{a}
	LC_{25}	7742.00 ± 29.44^{a}	8272.00 ± 117.26^{ab}	7854.50 ± 22.50^{a}
	LC_{30}	7807.00 ± 35.24^{a}	8697.00 ± 96.75^{a}	7914.50 ± 21.74^{a}
T4	CK	866.50 ± 27.88^{c}	886.50 ± 37.25^{c}	879.75 ± 38.08^{c}
	LC_{10}	1241.50 ± 14.54^{b}	1243.50 ± 13.36^{b}	1242.75 ± 15.61^{b}
	LC_{25}	1725.25 ± 4.73^{a}	1736.25 ± 4.61^{a}	1728.00 ± 2.68^{a}
	LC_{30}	1781.75 ± 2.50^{a}	1791.25 ± 3.35^{a}	1786.50 ± 2.78^{a}

T3 and T4 were measured after 7, 14, and 21 days of exposure to the solvent control (CK) or CLO at LC_{10} (15.35 mg a.i./L), LC_{25} (31.96 mg a.i./L), and LC_{30} (36.07 mg a.i./L). Values are presented as mean ± SE (n = 4). Different lowercase letters within the same measurement and time point indicate significant differences among treatments (one-way ANOVA with Tukey's post hoc test, $p < 0.05$). CK = solvent control.

Table S3. Locomotor performance of *F. cancrivora* tadpoles following CLO exposure (CK, LC_{10}, LC_{25}, LC_{30}) was measured at 24, 48, 72, and 96 h using the Video-Track system.

**Measurement**	**Concentration**	**Time (h)**			
		**24**	**48**	**72**	**96**
Total distance traveled (mm)	CK	271.57±35.60^{a}	241.48±32.65^{a}	252.05±28.72^{a}	282.62±30.94^{a}
	LC_{10}	212.77±32.47^{a}	199.67±39.87^{a}	146.83±34.57^{ab}	122.55±22.81^{b}
	LC_{25}	197.70±35.32^{a}	192.38±35.65^{a}	137.80±27.20^{b}	97.58±17.76^{b}
	LC_{30}	184.30±25.33^{a}	165.18±24.20^{a}	111.08±22.02^{b}	87.78±17.76^{b}
Average speed (mm/s)	CK	2.34±0.25^{a}	2.06±0.18^{a}	1.97±0.15^{a}	1.91±0.20^{ab}
	LC_{10}	2.22±0.14^{a}	1.79±0.07^{a}	2.00±0.11^{a}	2.17±0.15^{a}
	LC_{25}	2.48±0.08^{a}	2.00±0.17^{a}	1.82±0.11^{a}	1.80±0.11^{ab}
	LC_{30}	1.71±0.40^{a}	1.85±0.07^{a}	1.75±0.07^{a}	1.44±0.05^{b}

Distance traveled and swimming speed are reported as mean ± SE (n = 6 tadpoles per treatment per time point). Units follow the Video-Track output. Different lowercase letters within the same measurement and time point indicate significant differences among treatments (one-way ANOVA with Tukey's post hoc test, $p < 0.05$). CK = solvent control.

Table S4. Effects of CLO exposure on predation of BPH by *F. cancrivora* across developmental stages (G46–G48).

**Measurement**	**Concentration**	**Gosner stage**		
		**G46**	**G47**	**G48**
BPH consumed (No. / 24 h)	CK	32.75 ± 0.85^{a}	35.75 ± 0.48^{a}	37.00 ± 0.41^{a}
	LC_{10}	22.25 ± 1.25^{b}	24.25 ± 0.85^{b}	26.25 ± 0.48^{b}
	LC_{25}	18.00 ± 1.47^{c}	20.00 ± 0.41^{b}	21.75 ± 0.48^{c}
	LC_{30}	15.00 ± 0.40^{d}	18.25 ± 0.48^{c}	20.25 ± 0.25^{c}

Frogs were offered 50 live BPH for 24 h, and the number consumed was recorded. Values are mean ± SE (n = 4 frogs per treatment per developmental stage). Different lowercase letters within the same developmental stage indicate significant differences among treatments (one-way ANOVA with Tukey's post hoc test, $p < 0.05$). CK = solvent control.

References

1. Wang, Q., et al., *Exposure of zebrafish embryos/larvae to TDCPP alters concentrations of thyroid hormones and transcriptions of genes involved in the hypothalamic–pituitary–thyroid axis.* Aquatic toxicology, 2013. **126**: p. 207-213.
2. Chen, L., et al., *Prenatal transfer of polybrominated diphenyl ethers (PBDEs) results in developmental neurotoxicity in zebrafish larvae.* Environmental science & technology, 2012. **46**(17): p. 9727-9734.
